# Global Glomerulosclerosis and Segmental Glomerulosclerosis Could Serve as Effective Markers for Prognosis and Treatment of IgA Vasculitis With Nephritis

**DOI:** 10.3389/fmed.2020.588031

**Published:** 2020-10-23

**Authors:** Jiaxing Tan, Yicong Xu, Zheng Jiang, Gaiqin Pei, Yi Tang, Li Tan, Zhengxia Zhong, Padamata Tarun, Wei Qin

**Affiliations:** ^1^Division of Nephrology, Department of Medicine, West China Hospital, Sichuan University, Chengdu, China; ^2^West China School of Medicine, Sichuan University, Chengdu, China

**Keywords:** IgA vasculitis with nephritis, global glomerulosclerosis, segmental glomerulosclerosis, treatment, prognosis

## Abstract

**Background:** This study was aimed at investigating the clinical significance and curative effect of global glomerulosclerosis (GS) and segmental glomerulosclerosis (S) in adult-onset IgA vasculitis with nephritis (IgAV-N) patients since there was no consensus pathological grading method for adult IgAV-N.

**Methods:** A total of 188 biopsy-proven IgAV-N patients were prospectively identified. Patients were separately assigned to GS0/GS1/GS2 group and S0/S1/S2 based on the scores of global glomerulosclerosis and segmental glomerulosclerosis (0% /0–15% />15%, respectively).

**Results:** GS0, GS1, and GS2 occurred in 56.4, 29.2, and 14.4% of the adult-onset IgAV-N, respectively. Patients in GS2 group tended to have the most serious renal deterioration and the highest levels of blood pressure. IgAV-N patients were also divided into S0 group (64.4%), S1 group (20.7%), and S2 group (14.9%), where no obvious differences in baseline data were noted. K–M curves indicated that GS2 group had the worst renal outcome (*P* = 0.05) while there seemed to be no significant differences between GS0 group and GS1 group. In addition, no remarkable differences in primary outcome were found among S0 group, S1 group, and S2 group though the prognosis of S2 group tended to be the worst. However, the prognosis of S0/S1 group was markedly better than that of S2 (*P* = 0.04). The discrimination of poor prognosis could be improved by adding the pathological indicators of global glomerulosclerosis and segmental glomerulosclerosis. Most importantly, immunosuppressive treatment might be a superior alternative in IgAV-N patients without sclerosis scores or with lower level of sclerosis scores. But addition of immunosuppression was not recommended in patients with higher sclerosis scores.

**Conclusions:** Global glomerulosclerosis and segmental sclerosis might be used for management and treatment of adult-onset IgAV-N.

## Introduction

IgA vasculitis (IgAV), also named Henoch-Schönlein purpura, is a common form of systemic vasculitis that can cause abdominal pain, gastrointestinal bleeding, aching joints and renal damage. IgAV is a self-limited systemic disorder. But it can cause chronic kidney disease (CKD) when it affects the kidneys, which we name IgA vasculitis with nephritis (IgAV-N). Renal deterioration is the most serious complication of IgAV and is also a determinant factor of adverse prognosis (1). It has been acknowledged that proteinuria and hematuria cannot always reflect the renal damage accurately. Renal biopsy has high value for clinical decision-making and prognosis. Since IgAV-N was more common in children, few researches have investigated which pathological classification is suitable for adult-onset IgAV-N (2, 3). Therefore, a pathological classification for adult-onset IgAV-N that can be applied to predicting prognosis and guiding treatment, needs to be produced.

The guidelines published by the Kidney Disease: Improving Global Outcomes (KDIGO) indicated that angiotensin-converting-enzyme inhibitor (ACEI)/angiotensin receptor blockers (ARB) and corticosteroids were recommended for IgAV-N patients based on the clinical manifestations instead of pathological indicators, and the combination therapy of immunosuppressants and steroids is under debate (4–6). Emerging studies have demonstrated that the usage of immunosuppressants is significantly correlated to clinical remission of IgAV-N (7, 8). However, it is not clear exactly what kind of IgAV-N patients will benefit the most from the immunosuppressive therapy.

As a chronic progressive kidney disease, IgAV-N can result in end-stage renal disease (ESRD), in which chronic impairments such as chronic fibrosis and sclerosis are common. Theoretically, the appearance of global glomerulosclerosis and segmental sclerosis in kidney, which are typical chronic kidney injury, may predict a poor prognosis (9). However, few studies have proved it in the adult-onset IgAV-N. At the same time, few articles have proposed a targeted treatment option for the pathological types of IgAV-N. Hence, this study aims to elucidate the clinical significance of global glomerulosclerosis and segmental sclerosis on renal outcomes in adult patients with IgAV-N and to prescribe a course of treatment based on degree of renal sclerosis.

## Methods and Methods

### Subjects

We prospectively recruited 209 IgAV-N patients from October 2010 to June 2017 in West China Hospital, Sichuan University. A definitive diagnosis of IgAV-N was based on typical clinical manifestations and renal biopsies, according to the American College of Rheumatology (ACR) guidelines (10). The age of all individuals enrolled had to be more than 14 years old at the first onset of IgAV-N. The exclusion criteria included insufficient clinicopathological data for pathological classification (with <8 glomeruli in the renal biopsy sample) and/or other systemic diseases like diabetes, hepatitis, systemic lupus erythematosus (SLE), HIV infection and so on. All the participants were followed up regularly by reexaminations in our hospital for at least 6 months unless they reached the endpoint. Finally, 188 adult-onset IgAV-N patients were analyzed in the study. This was an observational study that was approved by the Ethics Committee of West China Hospital of Sichuan University. Participants kept informed by face-to-face interviews and written informed consent were obtained.

### Clinical Parameters and Treatments

The clinical indicators we recorded included symptoms and signs (edema, joint pain, abdominal pain and bloody stool), systolic blood pressure, diastolic blood pressure, proteinuria, urine red blood cell, serum albumin, serum creatinine, and estimated glomerular filtration rate (eGFR). Nephrotic syndrome was defined as massive proteinuria (>3.5 g/24 h) and hypoproteinemia (≤ 30 g/L). Hypertension was diagnosed as the resting blood pressure ≥140/90 mmHg.

The options of treatment modalities were determined by both the attending doctors and the patients, which was not interfered by the researchers since this was an observational study. Three commonly used treatments included supportive medical care with full dose angiotensin-converting-enzyme inhibitor (ACEI) or angiotensin receptor blockers (ARB), steroid therapy (0.5–1 mg/kg daily and tapered down within 6–8 months) with optimal dose of ACEI/ARB, and immunosuppressants (mycophenolate mofetil, cyclophosphamide, or azathioprine) combined with corticosteroids.

### Pathology Data and Groups

The pathological evaluation of this experiment was completely blind. If the patients with IgA vasculitis (purpura with or without abdominal pain, gastrointestinal bleeding, or aching joints) were manifested with hematuria, proteinuria and/or renal failure, the renal biopsy was performed by the supervising physicians. The renal biopsies were evaluated by a professional pathologist and an experienced clinician in our medical centers. All researchers did not participate in this pathological evaluation.

The pathological classification including mesangial proliferation (M0/M1, absent/present), endocapillary proliferation (E0/E1,absent/present),segmentalglomerulosclerosis (S), tubular atrophy or interstitial fibrosis (T0/T1,absent/present), and crescent injury (C0/C1,absent/present) was primarily based on the updated Oxford classification (3). Global glomerulosclerosis (GS) was also considered in the study and was defined as glomerular impairment with more than 50% of any one glomerulus manifested as scarring lesion or hyaline deposition (9). The definition of segmental glomerulosclerosis was sclerosis or adhesion in part but not the entire glomerulus, where capillary lumina were obliterated by matrix (11).

Previous studies have proved that IgA nephropathy (IgAN) patients with glomerulosclerosis > 25% of glomeruli have quite a bad prognosis (9). If it was grouped by 25% in our cohort, the distribution of patients was severely unbalanced, which was not suitable for reasonable statistical analysis. In order to increase the predictive sensitivity of this indicator, we grouped patients of different pathological types based on sample size and clinical experience, which was not exactly the same as Oxford classification. Segmental glomerulosclerosis was scored by percentages (S0/S1/S2, no segmental glomerulosclerosis/>0% of glomeruli but ≤ 15% of glomeruli/>15% of glomeruli). Patients with or without global glomerulosclerosis were divided into three groups (GS0/GS1/GS2, 0%/0–15%/>15% of glomeruli).

### Clinical Outcomes and Remission

The primary outcome consisted of end-stage renal disease (ESRD) defined as e-GFR <15 mL/min per 1.73 m2 or receiving maintenance renal replacement treatment, a 60% decline in the e-GFR and/or death. The secondary outcomes included complete remission (24 h-proteinuria ≤ 0.3 g, with no hematuria or impaired renal function); partial remission (24 h-proteinuria ≤ 1 g, with or without recurrent hematuria, or proteinuria decline > 50%); and no response (24 h-proteinuria >1.0 g or the decrease level of eGFR >10%).

### Statistical Analysis

Numbers with frequencies were used in statistical descriptions of nominal and grade variables while continuous variables were presented as mean ± standard deviation (homoscedastic) or median with interquartile ranges (heteroscedastic). Student's *t-*test, Wilcoxon test, ANOVA, or non-parametric Mann–Whitney *U-*test were selectively used to analyze continuous variables according to groups and data distribution. Pearson's chi-squared test or Fisher's exact test was adopted for categorical variables. Pearson correlation analysis or Spearman correlation analysis was used to explore the relationship among pathological indicators. Kaplan-Meier estimates was constructed to compute the proportions of endpoint in different groups and multivariate Cox regression analysis was established to identify the unfavorable factors for long-term renal outcome of IgAV-N, where hazard ratios (HRs), and confidence intervals (CIs) were used. Receiver operating characteristic (ROC) with area under curves (AUC) were inducted to measure the prediction accuracy. All tests were two-tailed and it was considered to be significant as the *p* < 0.05.

## Results

### Demographic and Clinical Characteristics

A total of 209 adult-onset IgAV-N patients diagnosed by clinical signs and renal biopsy were included in this study. Of them, 18 patients were excluded because of missing or the presence of other systemic diseases and 3 were excluded because of inadequate pathological biopsy specimens as <8 glomeruli in the renal biopsy sample. Accordingly, the study cohort finally consisted of 188 patients. The mean age was 30.9 ± 15.2 years at the time of biopsy. The follow-up time was 27.06 ± 20.09 months on average. Patients were further categorized into different groups according to glomerulosclerosis.

Grouped by global glomerulosclerosis, GS0, GS1, and GS2 occurred in 56.4, 29.2, and 14.4% of the adult-onset IgAV-N, respectively (Table 1). It was noted that global glomerulosclerosis scores increased with age (*P* < 0.01). The extrarenal symptoms, especially joint and abdominal involvement, were more prevailing in patients without global glomerulosclerosis (*P* < 0.01). Patients with higher rates of global glomerulosclerosis tended to have more serious renal deterioration and higher levels of blood pressure. Given that the differences in clinicopathological characteristics between GS0 and GS1 were relatively small, we considered GS0 and GS1 as a group GS0/GS1. Patients with lower scores of global glomerulosclerosis (GS0/GS1 group) seemed to have a milder illness with significantly higher level of eGFR, and lower levels of serum creatinine and blood pressure (both SBP and DBP).

**Table 1:** Clinicopathological manifestations of IgAV-N patients at baseline, grouped by the percentage of global glomerulosclerosis.

	**Global glomerulosclerosis**				**Global glomerulosclerosis**		
**Variables**	**GS0**	**GS1**	**GS2**	***P***	**GS0/GS1**	**GS2**	***P***
Numbers (%)	106 (56.4)	55 (29.2)	27 (14.4)	–	161 (85.6)	27 (14.4)	–
Male (%)	54 (50.9)	24 (43.6)	9 (33.3)	0.23	78 (48.3)	9 (33.3)	0.21
Age (years)	19.0 (16.0–38.3)	37.0 (21.0–50.0)	43.0 (22.0–55.0)	**<0.01**	29.5 ± 14.6	39.3 ± 16.8	**<0.01**
Interval from disease onset to biopsy (months)	1.0 (0.7–5.0)	2.0 (0.68–7.0)	4.5 (1.0–12.0)	0.13	2.0 (0.7–6.0)	4.5 (1.0–12.0)	0.06
**Clinical symptoms**							
Skin purpura (%)	96 (90.6)	54 (98.2)	23 (85.2)	0.09	150 (93.2)	23 (85.2)	0.24
Edema (%)	35 (33.0)	24 (43.6)	13 (48.1)	0.22	59 (36.6)	13 (48.1)	0.29
Abdominal pain (%)	38 (35.8)	10 (18.2)	5 (18.5)	**0.03**	48 (29.8)	5 (18.5)	0.26
Bloody stools (%)	17 (16.0)	7 (12.7)	0 (0)	**0.05**	24 (14.6)	0 (0)	**0.03**
Joint pain (%)	27 (25.5)	10 (18.2)	1 (3.7)	**0.03**	37 (23.0)	1 (3.7)	**0.02**
SBP (mmHg)	120.0 (110.0–128.3)	123.0 (113.0–135.0)	131.0 (115.0–155.0)	**<0.01**	120.0 (112.0–130.0)	131.0 (115.0–155.0)	**<0.01**
DBP (mmHg)	78.0 (70.8–85.0)	80.0 (71.0–86.0)	82.0 (75.0–95.0)	0.07	78.5 ± 11.7	84.1 ± 12.6	**0.02**
**Laboratory index**							
Proteinuria (g/24 h)	3.11 ± 3.05	3.03 ± 3.37	3.14 ± 2.61	0.98	3.08 ± 3.15	3.14 ± 2.61	0.93
Alb (g/L)	34.50 ± 8.34	36.35 ± 7.99	35.28 ± 7.39	0.39	35.13 ± 8.25	35.28 ± 7.39	0.93
sCr (umol/L)	64.45 (52.80–84.15)	71.00 (59.00–86.60)	88.00 (66.00–139.00)	**<0.01**	68.00 (56.80–84.95)	88.00 (66.00–139.00)	**0.02**
eGFR (ml/min/1.73 m2)	124.70 (94.35–134.78)	105.40 (87.30–116.80)	71.50 (50.90–110.00)	**<0.01**	109.05 ± 30.82	78.05 ± 38.21	**<0.01**
u–RBC (/HP)	184 ± 435	173 ± 427	180 ± 440	0.99	180 ± 431	180 ± 440	0.98
**Pathological features**							
M (%)	87 (82.1)	48 (87.3)	25 (92.6)	0.34	135 (83.9)	25 (92.6)	0.27
E (%)	24 (22.6)	7 (12.7)	1 (3.7)	**0.03**	31 (19.3)	1 (3.7)	**0.05**
S (%)	36 (34.0)	22 (40.0)	10 (37.0)	0.76	58 (36.0)	10 (37.0)	1.00
T (%)	22 (20.8)	31 (56.4)	23 (85.2)	**<0.01**	53 (32.9)	23 (85.2)	**<0.01**
C (%)	46 (43.4)	17 (30.9)	10 (37.0)	0.30	63 (39.1)	10 (37.0)	1.00
0	59 (55.7)	38 (69.1)	17 (63.0)		97 (60.3)	17 (63.0)	
0–25%	30 (28.3)	13 (23.6)	6 (22.2)		43 (26.7)	6 (22.2)	
>25%	17 (16.0)	4 (7.3)	4 (14.8)		21 (13.0)	4 (14.8)	
**Treatment**				**0.02**			**<0.01**
ACEI/ARB	6 (5.7)	4 (7.3)	7 (25.9)	**0.01**	10 (6.2)	7 (25.9)	**<0.01**
Steroids	39 (36.8)	25 (45.5)	11 (40.7)	0.57	64 (39.8)	11 (40.7)	0.54
Immunosuppressor	61 (57.5)	26 (47.3)	9 (33.3)	0.06	87 (54.0)	9 (33.3)	0.06

*IgAV-N, IgA vasculitis with nephritis; SBP, systolic blood pressure; DBP, diastolic blood pressure; ALB, albumin; sCr, serum creatinine; eGFR, estimated glomerular filtration rate; u-RBC, the count of uric red blood cell; M, mesangial proliferation; E, endocapillary proliferation; S, segmental glomerulosclerosis; C, crescents; T, tubular atrophy/interstitial fibrosis; GS, global glomerulosclerosis; ACEI, angiotensin-converting-enzyme inhibitor; ARB, angiotensin receptor blockers. The bold values mean statistically significant difference*.

IgAV-N patients were also divided into S0 group (64.4%), S1 group (20.7%) and S2 group (14.9%), based on the scores of segmental glomerulosclerosis (Table 2). No evident differences were observed in clinical manifestations and laboratory indexes except that patients without segmental glomerulosclerosis were more likely to suffer from gastrointestinal symptoms (*P* = 0.06). Similarly, S0 group and S1 group were treated as the same group (S0/S1). A relatively higher level of serum creatinine was found in patients in S2 group, compared with S0/S1 group (*P* = 0.06), while no other differences in baseline data were noted.

**Table 2:** Clinicopathological manifestations of IgAV-N patients at baseline, grouped by the percentage of segmental glomerulosclerosis.

	**Segmental glomerulosclerosis**				**Segmental glomerulosclerosis**		
**Variables**	**S0**	**S1**	**S2**	***P***	**S0/S1**	**S2**	***P***
Numbers (%)	121 (64.4)	39 (20.7)	28 (14.9)	–	160 (85.1)	28 (14.9)	–
Male (%)	56 (46.3)	15 (38.5)	16 (57.1)	0.33	71 (44.4)	16 (57.1)	0.23
Age (years)	31.2 ± 15.4	31.5 ± 15.6	28.5 ± 14.3	0.67	31.3 ± 15.4	28.5 ± 14.3	0.37
Interval from disease onset to biopsy (months)	7.4 ± 15.9	4.3 ± 5.2	5.9 ± 7.8	0.46	6.7 ± 14.1	5.9 ± 7.8	0.78
**Clinical symptoms**							
Skin purpura (%)	113 (93.4)	34 (87.2)	26 (92.9)	0.48	147 (91.9)	26 (92.9)	1.00
Edema (%)	50 (41.3)	14 (35.9)	8 (28.6)	0.44	64 (40.0)	8 (28.6)	0.30
Abdominal pain (%)	41 (33.9)	6 (15.4)	6 (21.4)	0.06	47 (29.4)	6 (21.4)	0.50
Bloody stools (%)	19 (15.7)	1 (2.6)	4 (14.3)	0.08	20 (12.5)	4 (14.3)	0.76
Joint pain (%)	25 (20.7)	11 (28.2)	2 (7.1)	0.10	36 (22.5)	2 (7.1)	0.08
SBP (mmHg)	123.8 ± 18.7	121.3 ± 19.9	126.1 ± 17.5	0.58	123.2 ± 19.0	126.1 ± 17.5	0.45
DBP (mmHg)	79.0 (71.5–85.0)	80.0 (71.0–95.0)	81.5 (70.3–94.8)	0.24	80.0 (71.0–85.0)	81.5 (70.3–94.8)	0.18
**Laboratory index**							
Proteinuria (g/24 h)	3.06 ± 3.28	3.22 ± 2.88	3.05 ± 2.41	0.96	3.10 ± 3.18	3.05 ± 2.41	0.94
Alb (g/L)	34.85 ± 8.19	36.37 ± 8.48	34.80 ± 7.33	0.58	35.22 ± 8.26	34.80 ± 7.33	0.80
sCr (umol/L)	78.52 ± 40.95	82.23 ± 39.21	95.81 ± 48.33	0.15	79.43 ± 40.44	95.81 ± 48.33	0.06
eGFR (ml/min/1.73 m2)	113.80 (89.20–129.70)	109.20 (80.70–132.00)	99.00 (64.13–130.47)	0.22	112.80 (87.58–129.88)	99.00 (64.13–130.47)	0.17
u-RBC (/HP)	193 ± 510	160 ± 254	150 ± 297	0.85	185 ± 460	150 ± 297	0.94
**Pathological features**							
M (%)	99 (81.8)	35 (89.7)	26 (92.9)	0.10	134 (83.8)	26 (92.9)	0.26
E (%)	25 (20.7)	6 (15.4)	1 (3.6)	0.08	31 (19.4)	1 (3.6)	**0.05**
T (%)	47 (38.8)	16 (41.0)	13 (46.4)	0.74	63 (39.4)	13 (46.4)	0.53
C (%)	53 (43.8)	11 (28.2)	9 (32.1)	0.16	64 (40.0)	9 (32.1)	0.29
0	67 (55.4)	28 (71,8)	19 (67.9)		95 (59.4)	19 (67.9)	
0–25%	31 (25.6)	11 (28.2)	7 (25.0)		42 (26.3)	7 (25.0)	
>25%	23 (19.0)	0 (0)	2 (7.1)		23 (14.4)	2 (7.1)	
GS (%)	50 (41.3)	22 (56.4)	12 (42.9)	0.26	72 (45.0)	12 (42.9)	0.84
**Treatment**				0.41			0.59
ACEI/ARB	14 (11.6)	1 (2.6)	2 (7.1)	0.27	15 (9.4)	2 (7.1)	1.00
Steroids	48 (39.7)	18 (46.2)	9 (32.1)	0.54	66 (41.3)	9 (32.1)	0.24
Immunosuppressor	59 (48.8)	20 (51.3)	17 (60.7)	0.53	79 (49.4)	17 (60.7)	0.18

*IgAV-N, IgA vasculitis with nephritis; SBP, systolic blood pressure; DBP, diastolic blood pressure; ALB, albumin; sCr, serum creatinine; eGFR, estimated glomerular filtration rate; u-RBC, the count of uric red blood cell; M, mesangial proliferation; E, endocapillary proliferation; C, crescents; T, tubular atrophy/interstitial fibrosis; GS, global glomerulosclerosis; ACEI, angiotensin-converting-enzyme inhibitor; ARB, angiotensin receptor blockers*.

### Pathological Findings

As shown in Table 1, the proportion of endocapillary proliferation were much lower in GS2 group (*P* = 0.03), whereas the proportion of tubular atrophy or interstitial fibrosis were extremely higher (*P* < 0.01), compared with GS0 group and GS1 group. Table 2 reveals the pathological findings of patients with different scores of segmental glomerulosclerosis and indicates that patients in S0/S1 group had higher rates of endocapillary proliferation (*P* = 0.05). Therefore, we speculated that there was a certain correlation between pathological impairments.

Then a Spearman correlation analysis was conducted (Table 3). It could be easily found that mesangial proliferation was positively related to segmental glomerulosclerosis and tubular atrophy/interstitial fibrosis (*r* = 0.16, *P* = 0.03). Endocapillary proliferation was inversely correlated to tubular atrophy/interstitial fibrosis and global glomerulosclerosis (*r* = –0.17, *P* = 0.02; *r* = –0.17, *P* = 0.01, respectively) while had a positive correlation with crescents (*r* = 0.22, *P* < 0.01). Most remarkably, global glomerulosclerosis and tubular atrophy/interstitial fibrosis had a strong correlation (*r* = 0.48, *P* = 0.01).

**Table 3:** Correlation analysis of pathological indicators.

**Mesangial proliferation (M)**					
*r* = 0.07, *p* = 0.34	**Endocapillary proliferation (E)**				
***r =* 0.16, *p =* 0.03**	*r* = −0.14, *p* = 0.07	**Segmental glomerulosclerosis (S)**			
***r =* 0.16, *p =* 0.03**	***r =* –0.17, *p =* 0.02**	*r* = 0.10, *p* = 0.17	**Tubular atrophy interstitial fibrosis (T)**		
*r* = 0.12, *p* = 0.11	***r =* 0.22, *p* < 0.01**	*r* = −0.12, *p* = 0.09	*r* = −0.01, *p* = 0.88	**CrescentLesions (C)**	
*r* = 0.11, *p* = 0.15	***r =* −0.17, *p =* 0.01**	*r* = 0.06, *p* = 0.43	***r =* 0.48, *p* < 0.01**	*r* = −0.10, *p* = 0.17	**Global glomerulosclerosis (GS)**

*The bold values mean statistically significant difference*.

### Renal Survival

**Figure 1:**
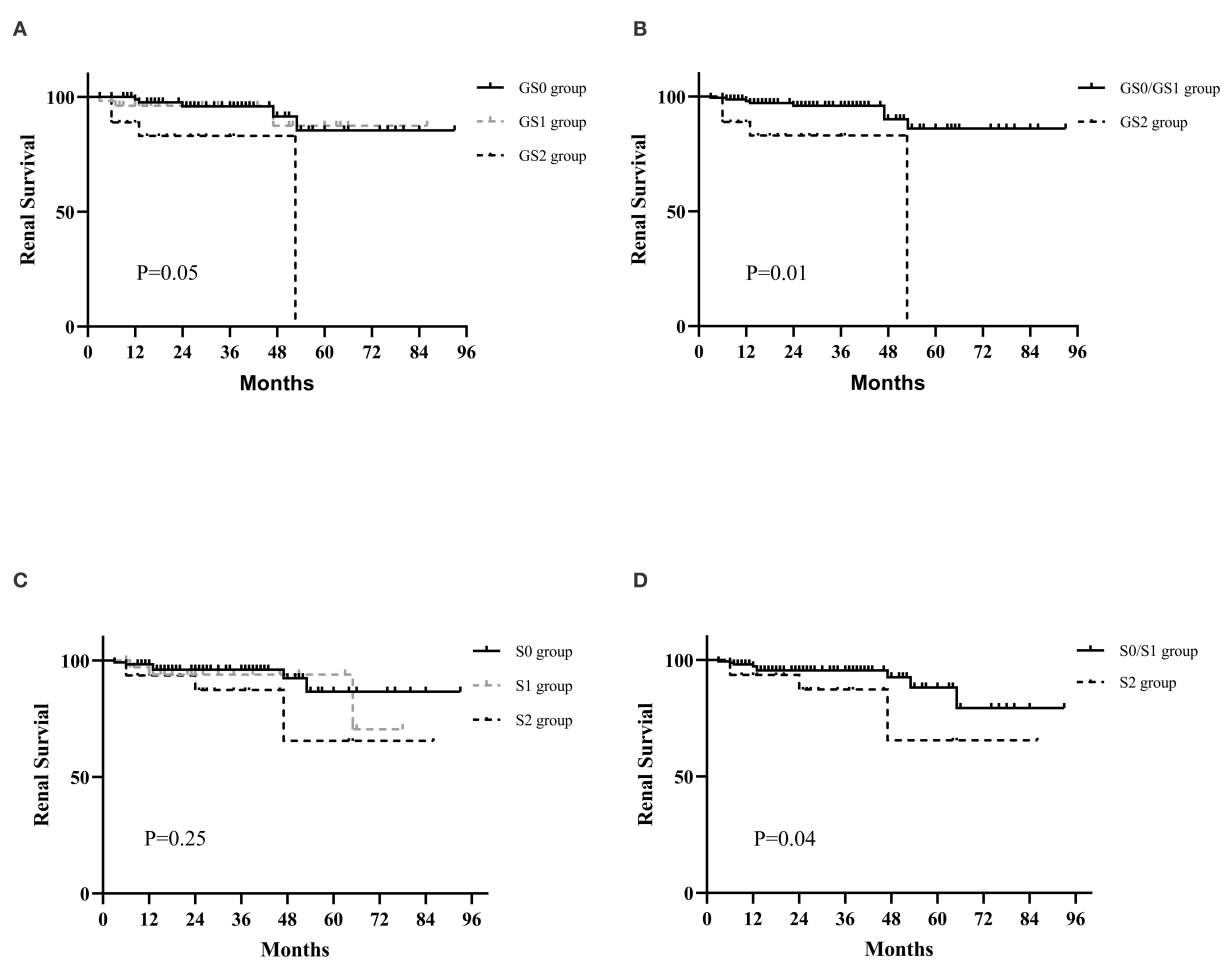
Kaplan-Meier curves for the probability of primary outcomes in IgAV-N patients with different global glomerulosclerosis and segmental glomerulosclerosis scores. **(A)** The renal prognosis of IgAV-N patients among GS0, GS1, and GS2 groups. **(B)** The renal prognosis of IgAV-N patients in GS0/GS1 and GS2 groups. **(C)** The renal outcomes of IgAV-N patients among S0, S1, and S2 groups. **(D)** The renal outcomes of IgAV-N patients in S0/S1 and S2 groups. GS0 group: Patients with 0% global glomerulosclerosis. GS1: Patients with >0 and ≤15% global glomerulosclerosis. GS2: Patients with >15% global glomerulosclerosis. GS0/GS1 group: Patients with ≥0 and ≤15% global glomerulosclerosis. S0 group: Patients with 0% segmental glomerulosclerosis. S1: Patients with >0 and ≥15% segmental glomerulosclerosis. S2: Patients with >15% segmental glomerulosclerosis. S0/S1 group: Patients with ≥0 and ≤15% segmental glomerulosclerosis.

A total of 13 patients finally reached the endpoint during their follow-up period and 9 of them progressed to ESRD. Figure 1 shows the renal survival based on classification of global glomerulosclerosis. It was worth noting that since no patients were followed for more than 50 months in GS2 group, the survival curve dropped sharply at 50 months. It was indicated that GS2 group had the worst renal outcome (Figure 1A, *P* = 0.05) while there seemed to be no significant differences between GS0 group and GS1 group. So, we merged the two groups and found that the renal survival of GS0/GS1 group was much better than that of GS2 group (Figure 1B, *P* = 0.01), indicating the higher the global glomerulosclerosis scores, the worse the prognosis.

The K-M survival analyses of IgAV-N divided by segmental glomerulosclerosis levels is also presented in Figure 1. In general, there were no significant differences in primary outcome among S0 group, S1 group, and S2 group though the prognosis of S2 group tended to be the worst (*P* = 0.25). However, the renal outcome of S0/S1 group was remarkably better than that of S2 (*P* = 0.04), suggesting that patients with segmental glomerulosclerosis more than 15% could predict the poor prognosis.

### Effects of S and GS on Discrimination of Prognosis

A multivariate COX regression model adjusted for Oxford classification, nephrotic syndrome, and treatment was carried out to evaluate the effects of global glomerulosclerosis and segmental glomerulosclerosis on prognosis. Notably, global glomerulosclerosis and segmental glomerulosclerosis could serve as independent predict markers adjusted for pathological indicators and partial clinical manifestations (HR 3.86, 95% CI 1.00–15.01, *P* = 0.05; HR 7.55, 95% CI 1.66–34.41, *P* = 0.01, respectively, Table 4). Moreover, other indicators of Oxford classification such as endocapillary proliferation and tubular atrophy/interstitial fibrosis, and nephrotic syndrome were also available for prognostic prediction.

**Table 4:** Prediction of renal outcomes in IgAV-N carried out by Cox-regression model.

	**Univariate analysis**		**Multivariate analysis**	
	**HR (95%CI)**	***p***	**HR (95%CI)**	***p***
GS2 (vs. GS1/GS2)	4.09 (1.21–13.90)	**0.02**	3.86 (1.00–15.01)	**0.05**
S2 (vs. S1/S2)	3.46 (1.03–11.57)	**0.04**	7.55 (1.66–34.41)	**0.01**
M1 (vs. M0)	2.04 (0.26–25.85)	0.49	0.97 (0.10–9.00)	0.98
E1 (vs. E0)	1.86 (0.55–6.32)	0.32	5.65 (1.04–30.70)	**0.05**
T1 (vs. T0)	3.28 (1.00–10.90)	**0.05**	8.23 (1.68–40.23)	**0.01**
C1 (vs. C0)	1.02 (0.32–3.26)	0.98	0.98 (0.28–3.50)	0.98
NS	5.76 (1.72–19.30)	**0.01**	11.32 (2.89–44.30)	**<0.01**
Immunosuppression	1.22 (0.39–3.85)	0.74	0.61 (0.17–2.14)	0.43

*GS, global glomerulosclerosis; S, segmental glomerulosclerosis; M, mesangial proliferation; E, endocapillary proliferation; T, tubular atrophy or interstitial fibrosis; C, crescents; NS, nephrotic syndrome. The bold values mean statistically significant difference*.

**Figure 2:**
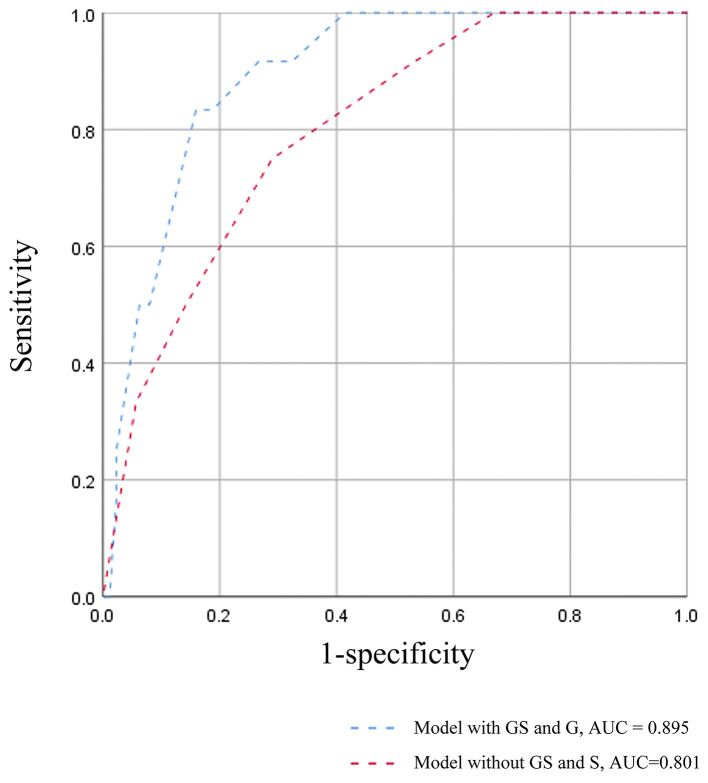
ROC curves for each model in prediction of the poor prognosis.

A survival model comprising the variables in the multivariate COX analysis, was established to further demonstrate the predictive power of global glomerulosclerosis and segmental glomerulosclerosis, which was measured by ROC curves (Figure 2). The AUC value of the survival model was 0.895. But when both global glomerulosclerosis and segmental glomerulosclerosis were removed, the AUC value dropped to 0.801. Accordingly, the discrimination of poor prognosis could be improved by adding the pathological indicators of global glomerulosclerosis and segmental glomerulosclerosis.

### Treatment and Response

Therapeutic schedules were displayed in relation to pathological classification (Tables 1, 2). Table 1 reveals that more patients in GS2 group were treated with optimal supportive care (25.9 vs. 6.2%, *P* <0.01) while immunosuppressive agents were less used (33.3 vs. 54.0%, *P* = 0.06). However, no obvious differences in treatment regimens among S0 group, S1 group, and S2 group (*P* > 0.10).

Considering that the use of immunosuppressants in IgAV-N was highly controversial, we analyzed whether the addition of immunosuppressants to routine treatment was beneficial to the clinical remission of patients with IgAV-N. Based on pathological lesions, the responses to each therapy were assessed in Table 5. IgAV-N patients receiving steroid combined with immunosuppressant therapy in GS0/GS1 group had a distinctly higher rate of complete remission (35.6 vs. 17.6%, *P* = 0.01). Nevertheless, immunosuppression seemed to have no benefit for complete remission in patients with higher scores of global glomerulosclerosis (33.4 vs. 27.8%, *P* = 1.00). In addition, the differences in the rate of no response between immunosuppressive therapy and treatment without immunosuppression could not be distinguished in either GS0/GS1 group or GS2 group.

**Table 5A:** The efficacy of immunosuppressants on clinical remissions of IgAV-N patients with different glomerulosclerosis scores.

	**GS0/GS1**			**GS2**			**S0/S1**			**S2**		
**Response**	**non-IT**	**IT**	***P***	**non-IT**	**IT**	***P***	**non-IT**	**IT**	***P***	**non-IT**	**IT**	***P***
CR (%)	13 (17.6)	31 (35.6)	0.01	5 (27.8)	3 (33.4)	1.00	16 (19.8)	26 (32.9)	0.07	2 (18.2)	8 (47.1)	0.12
PR (%)	46 (62.2)	36 (41.4)	0.01	9 (50.0)	4 (44.4)	1.00	47 (58.0)	34 (43.0)	0.08	8 (72.7)	6 (35.3)	0.12
NR (%)	15 (20.2)	20 (23.0)	0.41	4 (22.2)	2 (22.2)	1.00	18 (22.2)	19 (24.1)	0.85	1 (9.1)	3 (17.6)	1.00

*GS, global glomerulosclerosis; S, segmental glomerulosclerosis; CR, complete remission; PR, partial remission; NR, no response; IT, treatment with immunosuppressants; non-IT, treatment without immunosuppressants*.

Similar results were also found in S0/S1 group and S2 group. The immunosuppressants were more beneficial for patients in S0/S1 group because the complete remission rate was higher (32.9 vs. 19.8%, *P* = 0.07) whereas the addition of immunosuppressors seemed to be unnecessary in patients in S2 group.

Considering that it was unclear if the response to immunosuppression was just based on less chronic disease or the association with more endocapillary hypercellularity, further analysis was carried out. We found that patients without endocapillary hypercellularity in both GS0/GS1 and S0/S1 groups had a better response to immunosuppression agents because of the higher complete remission rate (Table 5B). However, for those with endocapillary hypercellularity but no chronic lesions (GS and S), the superiority of immunosuppressive agents did not seem obvious, indicating that sclerosis scores might have guiding significance for the choice of treatment. Therefore, it might be recommended that these two indicators be added to the pathological score of IgAV-N.

**Table 5B:** The efficacy of immunosuppressants on clinical remissions of IgAV-N patients with different glomerulosclerosis scores and endocapillary proliferation.

	**GS0/GS1 and E0**			**GS0/GS1 and E1**			**S0/S1 and E0**			**S0/S1 and E1**		
**Response**	**non-IT**	**IT**	***P***	**non-IT**	**IT**	***P***	**non-IT**	**IT**	***P***	**non-IT**	**IT**	***P***
CR (%)	10 (15.6)	24 (36.4)	0.01	3 (30.0)	7 (33.3)	1.00	13 (18.3)	19 (32.8)	0.06	3 (30.0)	7 (33.3)	1.00
PR (%)	44 (62.0)	27 (40.9)	<0.01	2 (20.0)	9 (42.9)	0.26	45 (63.4)	25 (43.1)	0.02	2 (20.0)	9 (42.9)	0.26
NR (%)	10 (15.6)	15 (22.7)	0.30	5 (50.0)	5 (23.8)	0.22	13 (18.3)	14 (24.1)	0.42	5 (50.0)	5 (23.8)	0.22

*GS, global glomerulosclerosis; S, segmental glomerulosclerosis; CR, complete remission; PR, partial remission; NR, no response; IT, treatment with immunosuppressants; non-IT, treatment without immunosuppressants*.

## Discussion

The International Study of Kidney Disease in Children (ISKDC) classification is the most commonly used histological classification for pediatric patients with IgAV-N and it was shown to be connected with long-term prognosis (2, 12, 13). Due to the heavier pathological damage and poorer outcomes in adults, ISKDC seems to be not sensitive enough to predict the prognosis of adult-onset IgAV-N (10, 14, 15). Since IgAV-N was more common in children, few researches have investigated which pathological classification is suitable for adult-onset IgAV-N. Emerging studies have demonstrated that the indicators of the Oxford classification of IgAN, which shared the similar pathophysiological mechanism with IgAV-N, can indicate the renal outcomes of IgAV-N (14, 16–19). A portion of scholars noted that they could be recommended for clinical practice. Unfortunately, because of the heterogeneity of the research results, this doctrine has not been widely accepted yet. This study was carried out to provide a novel pathological grading scheme to further refine the Oxford classification for adult-onset IgAV-N, which could better predict prognosis and guide treatment.

Our study noted that global glomerulosclerosis (>15% of glomeruli) and segmental glomerulosclerosis (>15% of glomeruli) had a significant correlation with poor prognosis, where more than 3- and 7-fold increased risk were observed, implying that global glomerulosclerosis and segmental glomerulosclerosis could be used as independent prognostic factors.

Global glomerulosclerosis is a kind of the nephrosclerosis that is often regarded as a chronic change. Besides patients with CKD, the kidneys of normal healthy aging may also show signs of nephrosclerosis (20). But the degree of global glomerulosclerosis seems to be more severe in patients with CKD. Several studies have demonstrated that global glomerulosclerosis could predict a worse outcome in patients with IgAN or other CKD (9, 20, 21). However, the role of it remains unknown in IgAV-N since no studies have been performed. In keeping with the above studies, it was uncovered in this study that global glomerulosclerosis was associated with an increased risk of adverse renal outcomes in adult IgAV-N, especially global glomerulosclerosis >15% of glomeruli. Notably, we also found that global glomerulosclerosis was positively correlated to tubulointerstitial atrophy and fibrosis, consistent with the previous study (22). Further Cox-regression analysis adjusted for clinical symptoms and pathological indicators were performed, and it should be noted that global glomerulosclerosis was an independent predictor (R 3.86, 95% CI 1.00–15.01, *P* = 0.05). Hence, it made a sense to add global glomerulosclerosis to pathology score system of adult IgAV-N.

Segmental glomerulosclerosis was a typical index of Oxford Classification of IgA nephropathy (IgAN), which has been widely accepted to identify the prognosis of IgAN (3, 11, 23). It has been reported that segmental glomerulosclerosis, as a more chronic stage of glomerulonephritis, may develop from the organization of endocapillary inflammatory lesions and/or segmental necrosis, which may lead to sclerosis of kidneys (3). Additionally, segmental glomerulosclerosis may be typically related to podocytopathies (24). Therefore, these could explain why IgAV-N patients with higher rate of segmental glomerulosclerosis had a poorer outcome in our study. However, whether segmental glomerulosclerosis can be used as a prognostic factor of IgAV-N does not reach a consensus. Some studies have proposed that segmental glomerulosclerosis is useful in predicting long-term renal outcome of IgAV-N (16, 19). But K-M analysis in other studies have indicated that it fails to predict the prognosis (25, 26). We speculated that the causes of the inconsistent conclusions may result from the enrolled patients with different severity of segmental glomerulosclerosis. What is noteworthy is that the mainstream classification of segmental glomerulosclerosis scores was the presence or absence (14, 16, 19, 25–28). Patients with higher proportion (>15%) and lower proportion (0–15%) of segmental glomerulosclerosis scores were assigned to the same group in their study. But our study found that the clinicopathological characteristics and prognosis of patients in S0 group and S1 group were similar. Therefore, distinguishing the rate of segmental glomerulosclerosis is more meaningful than distinguishing the presence or absence of it, which could be partially referred to T scores in Oxford Classification (11). In addition, the combination of global and segmental glomerulosclerosis could improve the effect power of survival model (Figure 2), which was reasonable to apply it in the clinic.

Previous studies have revealed that the combination treatment of corticosteroids and immunosuppressants has an advantage on IgAV-N remission (7). But unfortunately, the optimal indications for combination therapy have not been well-identified. Results of our study demonstrated that the response to immunosuppression differed greatly, depending on the severity of sclerosis. A higher complete remission rate for combination therapy were found in GS0/GS1 and S0/S1 groups, suggesting that immunosuppressants may be a superior alternative in treating HSPN without chronic changes or with relatively lower level of chronic changes. But for patients with higher scores of global and segmental glomerulosclerosis, adding immunosuppressor to corticosteroids was not necessary because there was no marked difference in response between the two regimens. Thus, global glomerulosclerosis and segmental glomerulosclerosis could serve as effective markers for prognosis and treatment of adult-onset IgAV-N.

There were some limitations of our study. Firstly, this was a single center study with limited sample size and the number of patients in GS2 and S2 group was relatively small. Secondly, the average follow-up time was nearly 30 months that was not long enough and that should be continuous. Thirdly, this was an observational study and no intervention was involved. There was no doubt that the baseline characteristics could not be fully controlled, which might result in potentially biased evaluation of efficacy.

## Conclusion

Global glomerulosclerosis and segmental sclerosis were independent risk factors of adverse outcome. Immunosuppressive treatment seemed to be a superior alternative in IgAV-N patients without sclerosis scores or with lower level of sclerosis scores. But addition of immunosuppression was not recommended in patients with higher sclerosis scores. Therefore, global glomerulosclerosis and segmental sclerosis might be used for management and treatment of adult-onset IgAV-N.

## Data Availability Statement

The raw data supporting the conclusions of this article will be made available by the authors, without undue reservation.

## Ethics Statement

The studies involving human participants were reviewed and approved by the Ethics Committee of West China Hospital of Sichuan University. Written informed consent to participate in this study was provided by the participants' legal guardian/next of kin.

## Author Contributions

WQ and JT: research idea, study design, and data analysis. JT, YX, ZJ, LT, YT, and ZZ: patient enrollment. JT and ZZ: clinical data collection. JT, YX, and PT: data acquisition. JT, YX, and WQ: statistical analysis. All authors contributed to the article and approved the submitted version.

## Conflict of Interest

The authors declare that the research was conducted in the absence of any commercial or financial relationships that could be construed as a potential conflict of interest.
