# Supplementary material for: Global Glomerulosclerosis and Segmental Glomerulosclerosis Could Serve as Effective Markers for Prognosis and Treatment of IgA Vasculitis With Nephritis
Source: Front Med (Lausanne). 2020 Oct 23;7:588031. doi: 10.3389/fmed.2020.588031 (PMC7646400; doi:10.3389/fmed.2020.588031)

	M0	M1	p	E0	E1	p	T0	T1	p
number	30	158		157	31		113	75	
age	27.8±15.3	31.6±15.5	0.221	30.7±15.2	31.8±17.2	0.759	28.65±18.83	34.59±15.95	0.01*
gender (%)	11/19	75/83	0.276	69/88	17/14	0.266	50/63(44.2/55.	36/39(48.0/52.0)	0.613
edema (%)	11(36.7)	60(38.0)	0.892	56(35.7)	15(48.4)	0.182	27.89±20.96	25.80±18.77	0.486
joint pain (%)	6(20.0)	32(20.3)	0.975	31(19.7)	7(22.6)	0.719	34(30.1)	37(49.3)	0.008*
abdominal pain (%)	9(30.0)	45(28.5)	0.866	40(25.5)	14(45.2)	0.027*	29(25.7)	9(12.0)	0.022*
bloody stool	3(10.0)	22(13.9)	0.771	18(11.5)	7(22.6)	0.096	43(38.1)	11(14.7)	0.001*
SBP	122.3±14.9	123.9±19.3	0.669	122.8±17.9	128.1±21.7	0.151	21(18.6)	4(5.3)	0.009*
DBP	78.6±9.9	79.4±12.3	0.723	79.4±12.4	78.9±8.9	0.862	118(109-129)	125(116-141)	<0.001*
hypertension	6(20.0)	40(25.3)	0.535	36(22.9)	10(32.3)	0.27	79.90±12.19	81.42±11.20	0.047*
proteinuria	2.7±4.3	3.1±2.8	0.609	2.00	2.4(1.4-6.0)	0.099	22(19.5)	24(32.0)	0.045*
u-RBC	115.2±218.6	197.9±474.3	0.351	34(9-134.5)	44(15-307)	0.22	2.91±2.71	3.20±3.49	0.521
ALB	38.3±9.6	34.7±7.8	0.027*	35.8±8.1	32.6±8.2	0.045	44(10-226)	31(8-110)	0.014*
Scr	63.4	71.1	0.045*	80.6±41.3	88.9±45.9	0.317	36.1(28.05-	37.10(32.50-	0.117
e-GFR	113.8±28.9	102.7±34.8	0.103	105.3±33.6	100.3±36.6	0.45	76.15±39.50	90.62±44.53	0.020*

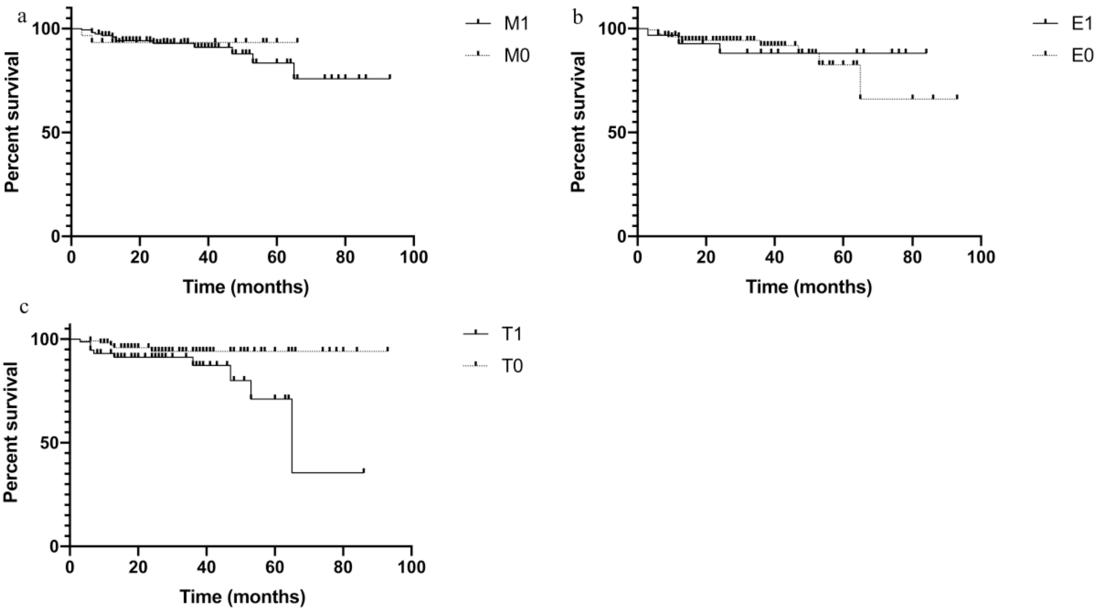

Supplement: Supplementary file 1 [file Data_Sheet_1.PDF]
